# Management of Cow’s Milk Allergy from an Immunological Perspective: What Are the Options?

**DOI:** 10.3390/nu11112734

**Published:** 2019-11-11

**Authors:** Edward F. Knol, Nicolette W. de Jong, Laurien H. Ulfman, Machteld M. Tiemessen

**Affiliations:** 1 Center Translational Immunology, University Medical Center Utrecht, 3584 CT Utrecht, The Netherlands; 2 Department of Internal Medicine, Section Allergology and Clinical Immunology, Erasmus MC, 3000 CA Rotterdam, The Netherlands; n.w.dejong@erasmusmc.nl; 3 FrieslandCampina, 3818 LE Amersfoort, The Netherlands; Laurien.Ulfman@frieslandcampina.com; 4 Danone Nutricia Research, 3584 CT Utrecht, The Netherlands; Machteld.TIEMESSEN@nutricia.com; 5 Institute of Pharmaceutical Sciences, Utrecht University, 3584 CG Utrecht, The Netherlands

**Keywords:** allergy, cow’s milk, formula, therapy, immune cells, Immunoglubuline E

## Abstract

The immunological mechanism underlying Immunoglobuline E (IgE)-mediated cow’s milk allergy has been subject to investigations for many years. Identification of the key immune cells (mast cells, B cells) and molecules (IgE) in the allergic process has led to the understanding that avoidance of IgE-crosslinking epitopes is effective in the reduction of allergic symptoms but it cannot be envisioned as a treatment. For the treatment and prevention of IgE-mediated cow’s milk allergy, it is thought that the induction of a sustained state of immunological tolerance is needed. In this review, we will discuss various approaches aimed at achieving immunological tolerance and their success. Furthermore, we will speculate on the involved immunological mechanism.

## 1. Immunological Aspects of Cow’s Milk Allergy

Of all known food allergies in infancy, cow’s milk allergy (CMA) is of special interest to immunologists as most allergic infants will acquire spontaneous tolerance toward cow’s milk before the age of 3 years. A Danish birth cohort showed that children with confirmed cow’s milk allergy appeared to be tolerant in 56% and 77% of the children at age 1 and 2 years, respectively [1,2]. While the incidence of cow’s milk allergy is estimated to be around 2–3%, less than 0.5% of adults suffer from CMA [3]. Ingestion of cow’s milk can lead to acute cutaneous symptoms, such as urticaria, and may also lead to immediate-type pulmonary and/or gastro-intestinal symptoms or, especially at older ages, systemic anaphylaxis [4]. Therefore, there is a need for proper allergy management during the “allergic” years and, in addition, options to accelerate the process of tolerance acquisition would be welcomed by affected families. Besides allergic infants, there is also a population of adults displaying Immunoglobuline E (IgE)-mediated cow’s milk allergy [5]. Cow’s milk protein (CMP) contains various proteins, of which the whey proteins—β-lactoglobulin and α-lactalbumin, and the caseins (αs1-, αs2-, β-, and κ-casein)—are the most important proteins regarding allergy. Specific IgE antibodies to all the subfractions of both casein and whey proteins can be detected in infants and children with CMA [5]. Besides B cell activity markers, such as IgE antibodies, T cell activity toward the various cow’s milk proteins (both whey proteins and caseins) can also be found. Of interest, CM-specific T cells can be isolated from both allergic and non-allergic individuals [6]. Upon T cell activation, the cytokine profile of a T cell will influence the subsequent B cell responses. B cells will class switch their immunoglobulin production under the influence of T-cell-derived cytokines. T-cell-derived Interleukin (IL)-4 production will cause the process of Ig class switching of B cells toward IgE (causing sensitization and subsequent allergy after exposure to the specific allergen), whereas IL-10 promotes the production of Immunoglobuline G4 (IgG4) (possibly involved in the process of immune tolerance). It has been demonstrated that cow’s-milk-specific IgG4 is linked to tolerance in children with increased levels of IgE [7,8]. However, there is still a scientific debate whether increased IgG4 might be just an indicator for increased/high exposure. Therefore, the preceding T cell response in cow’s-milk-allergic individuals will have a large impact on the immune response toward the proteins in cow’s milk, and thereby, the skewing towards an allergic or a tolerogenic response. T cells recognize specific parts of proteins (so called T cell epitopes) only when presented by antigen-presenting cells in the context of an human leucocyte antigen (HLA) molecule. Due to the large variation in genetic profiles between individuals (variation in HLA genotype), many different T cell epitopes may induce an immune response. Within some of the cow’s milk proteins, several dominant antigenic regions have been identified. For example, within αs1-casein and β-lactoglobulin, T cell epitopes have been identified [9,10]. Since these T cell epitopes appear to be recognized by T cells of both allergic and non-allergic donors, this suggests that these parts of the protein may potentially be involved in the process of tolerance induction. 

Importantly, the size of major histocompatibility complex (MHC) class II presented peptides is only 15–24 amino acids (aa) long, while the size of a protein that can crosslink IgE on mast cells or basophils is much larger, about 5–25 nm [11]. Therefore, as the T cell epitopes have the potential to steer the immune response without causing the detrimental process of IgE crosslinking on mast cells and/or basophils causing the allergic reaction, active immunotherapy using T cell epitopes/peptides may be an attractive option for cow’s-milk-allergic patients (both as a preventive as well as a therapeutic approach). One of the challenges is to identify enough diverse numbers of T cell epitopes, which are recognized by the majority of the target population, in order to provide enough stimulation of the immune system and to “re-train” the immune system away from an allergic response toward a tolerogenic response. As mentioned earlier, the HLA genotype diversity of the population will be of importance in the design of the diversity of the peptide mix needed to induce tolerance, as was demonstrated before for birch pollen allergens [12].

For αs1 and β caseins, it has been shown that the IgE binding epitopes are more in the 3D configuration in young children, whereas in adolescents and adults, it is more the linear structure that is recognized [13,14]. Remarkably, this was not the case for the IgG binding epitopes [14]. This might be related to the more immature digestive tract in young children, in which, for instance, the pH of the stomach is somewhat higher than later in life, leaving the more 3D configuration intact [15].

## 2. Cow’s Milk Formula, Including Hydrolysate in Cow’s-Milk-Allergic Patients

For infants that cannot be breastfed, other nutritional solutions (in the form of formula) are available. Several different cow’s-milk-protein-based formula are currently on the market, varying from a formula based on whole cow’s milk protein (CMP), partially hydrolyzed CMP, to extensively hydrolyzed CMP, and even amino-acid-based formulas. Extensively hydrolyzed cow’s milk formulas with documented hypoallergenicity are being recommended as a first-choice formula for cow’s-milk-allergic infants and young children [16,17]. It must be realized that this is mostly management, but not a cure, of the disease; therefore, in the future, interventions are needed that aim at preventing or curing CMA. For prevention, tolerance inducing partially hydrolyzed formulas have been developed for their potential to reduce the risk of CMA since these formulas contain peptides of a specific length. So far, contradictory results have been reported on the effect of these formulas on prevention. The largest individual study so far did find a lower risk on allergic manifestations, but in a recent meta-analysis, no evidence for a reduction in allergic manifestations (including CMA) was shown [18]. 

Factors, such as the selection of subjects, set up of study, type of product (hydrolysis degree, type of hydrolyzed cow’s milk protein), and studies performed in small groups of patients, could play a role in the observed discrepancies between studies. Furthermore, since the incidence of IgE-mediated CMA is low, and spontaneous tolerance occurrence is relatively high, a preventive study with a primary outcome on CMA (instead of all allergic manifestations) would be important but very challenging to conduct.

Recently, different commercially available infant formulas (intact proteins versus partially hydrolyzed versus extensively hydrolyzed) were investigated on their in vitro immune profile. Not all extensively hydrolyzed formulas reacted in a similar way with respect to IgE reactivity, proliferative responses of immune cells, and cytokine profiles [19]. This was also true for the partial hydrolysates. Direct comparison of in vivo reactivity of the hypoallergenic formulas in an at risk population and/or in CMA patients with immune profiles in these target groups is currently lacking in the literature, but would be needed to investigate whether in vitro profiles can explain the potential differences in effectiveness mechanistically.

By comparing the immune response toward whole protein cow’s milk formulas versus partial and extensive hydrolysates, it has been demonstrated that hydrolyzation of proteins reduces allergenicity whilst maintaining immunogenicity (T cell reactivity), depending on the type of hydrolysate [20,21]. However, the degree of hydrolysis not only corresponds to reduced allergenicity, it may also lead to reduced or different T cell reactivity [20,21]. The discrepancy between T cell activation profiles in these studies may be explained by different patient populations included in these studies. The serum and T cells used in the two studies were derived from cow’s-milk-allergic children [20] or adults [21], which may have an important impact on IgE binding to conformational versus linear epitopes [13,14]. As the cytokine profile is essential for the subsequent immunological processes, it is important to analyze the capacity of the different T cell epitopes in the different cow’s-milk-based formulas with regard to their immunostimulatory capacity. 

Another important approach is to consider unprocessed, raw cow’s milk instead of the commercially available heated and processed cow’s milk. Epidemiological data indicate that consumption of raw milk in the first year of life protects against allergies, including asthma [22]. There are several potent immunomodulating activities in milk that are lost during processing, such as transforming growth factor Bèta (TGF-β), IL-10, vitamin D, and lactoferrin, as well as fatty acids, oligosaccharides, and lipids, some of which are lost during processing [23]. Importantly, these bovine products have potent interspecies effects on human cells. However, although there are several potential advantages to introducing raw milk into the diet of young children, there is a significant risk of bacterial infections that also hampers controlled studies in infants to prove its effect [24]. Therefore, minimal processing techniques of raw milk to preserve the immunomodulating activity but safeguard the microbiological quality are needed. Alternatively, isolating the immune factors that drive the protective effects and provide these isolated components to infants is an interesting way to go.

Additionally, the composition of the intestinal microbiota might influence the immune response toward cow’s milk proteins (and the different T cell epitopes). Since it has been shown that the composition of the microbiota affects the development of the mucosal immune system, it is highly likely that differences in microbiota will shape the microenvironment in which the immune response is elicited [25]. Indeed, the microbiome of atopic versus healthy infants was shown to be different, and with specific pre- and/or probiotics, this dysbiosis may be altered toward a beneficial microenvironment in which tolerance toward cow’s milk proteins can be induced [26]. Interestingly, combining an extensively hydrolyzed formula with a probiotic strain may further accelerate tolerance development [27]. 

## 3. Baked Milk in the Treatment of Cow’s Milk Allergy

It has been suggested that the introduction of baked milk into the diet of the child may speed up the resolution of cow’s milk allergy [28]. Most children outgrow their cow’s milk allergy by the age of 3 years old [29]. These individuals with transient cow’s milk allergy produce IgE antibodies that are primarily directed at conformational epitopes. Since high temperatures (baking) reduce allergenicity by destroying these epitopes, the hypothesis is that the transient allergic group of children would tolerate baked milk products. More importantly, the addition of baked-milk products to the diet of these children appears to markedly accelerate the development of tolerance to unheated milk compared to a strict avoidance diet, which is currently the “standard of care” [28]. This approach is being introduced into clinical practice, although the hard evidence to underpin it seems to be lacking. 

In a recent systematic review by Lambert et al. to examine the evidence as to whether baked milk introduction into the diet leads to a larger proportion of children outgrowing their milk allergy, only three studies could be included [30]. Although the results are promising, e.g., baked milk was found to be well tolerated in children and no serious adverse reactions were reported, without randomization of the intervention, these studies are at a major risk of confounding by factors that are not equally distributed between the different groups. 

Nevertheless, introduction of baked milk into the diet can also increase the quality of life by expanding the diet, boosting nutrition, and promoting participating in social activities [31]. 

The study of potential biomarkers to predict the tolerability of baked milk, such as allergen-specific IgE or the skin prick test (SPT), are ongoing. A cow’s-milk-specific IgE level ≥15 KU_A_/L and SPT ≥8 mm in children ≤2 years old are highly predictive for a positive oral challenge reaction with baked milk [32]. Using specific IgG4 levels in addition to specific IgE levels may help predict baked milk reactivity. Casein and beta-lactoglobulin-specific IgE/IgG4 ratios appear to be significantly higher in baked-milk-reactive subjects compared to baked-milk-tolerant subjects [33].

Although there are currently no results of randomized controlled studies to determine whether baked milk speeds up the resolution of cow’s milk allergy, the opportunity to reduce the child’s dietary restrictions can potentially have a major beneficial effect on the food allergic child and their family. 

## 4. Allergen Immunotherapy in the Treatment of Cow’s Milk Allergy

A more direct approach to reach tolerance in the cow’s milk allergic patients is via allergen immunotherapy (AIT), in which cow’s milk is administered for a longer period, starting with micrograms, increasing to milligrams, and finally to grams via either the oral route, including sublingual administration [34], or epicutaneous route [35]. Each of these approaches is intended to induce some level of desensitization with repeated exposure to the allergenic food protein, although the risks and potential benefits of each treatment differ significantly [36]. Permanent tolerance is defined as the ability to ingest food without symptoms despite prolonged periods of avoidance or irregular intake [37]. Both intact cow’s milk, as well as partially hydrolyzed cow’s milk, has been tested. In a meta-analysis, it was shown that the relative risk for desensitization after allergen immunotherapy (AIT) for cow’s milk was 0.12, 95% CI = 0.06–0.25 [34]. At the same time, the safety of AIT is an issue and side effects are found frequently, though merely local [38]. Individual studies have evaluated the immunological parameters changed by AIT. After the treatment with partially hydrolyzed formula of cow’s-milk-allergic children and young adults (age range 1–20), a slight reduction in casein-specific IgE was demonstrated after 16 weeks, but no increased concentrations of IgG4, nor changes in casein-induced basophil degranulation [39]. In a younger group (7–12 months) treated with increasing cow’s milk concentrations, cow’s-milk-specific IgG4 increased 20–40-fold, while IgE decreased about 2-fold [8]. In a cohort of 2-year-old children, AIT strongly reduced the skin reactivity for cow’s milk and slightly increased (3-fold) the specific IgE concentrations for caseins and total cow’s milk [40]. Comparable levels of IgE decrease and IgG4 increase were noted in a study with older children (5–15 years). However, only casein-specific IgE decreased, but not IgE levels for α-lactalbumin and β-lactoglobulin [41]. Overall, even with the limited number of controlled studies, it seems that the ratio of specific IgE/IgG4 decreased, mostly by the increased amounts of IgG4. The clinical benefit of AIT in cow’s-milk-allergic patients is difficult to interpret, because this is only performed at a young age in which the incidence of spontaneous tolerance is significant [1]. Moreover, whereas the adult population needs a solution for cow’s milk allergy (CMA), there are no controlled studies performed with AIT in this age group and is therefore is not recommended in adults [38]. 

One of the challenges in the AIT for cow’s milk allergy is that clinical tolerance only remains when AIT is continued [34]. In contrast to AIT for insect venoms or pollen allergens, clinical tolerance to food allergens is lost rapidly after stopping the AIT procedure. The future challenge is therefore to induce sustained clinical tolerance after AIT is stopped.

## 5. Which Immune Cells Are Involved in Immunotherapy for Cow’s Milk Allergy?

**Figure 1:**
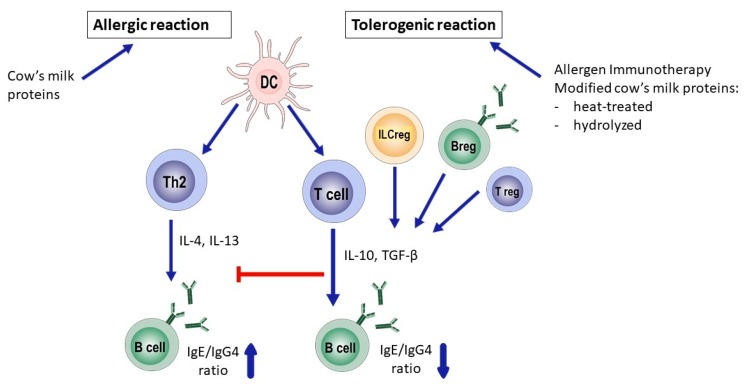
Immune response in the allergic versus tolerant state for cow’s milk proteins. Breg: B-regulatory cell; DC: Dendritic cell; ILCreg: Regulatory innate lymphoid cells; IL: interleukin; TGF- β: transforming growth factor Bèta; IgG4: Immunoglobulin G 4; IgE: immunoglobulin E; Treg: T regulatory cell; Th2: T- helper 2 cell.

To gain insight in the immunological mechanism of tolerance induction, the analysis of various immune cells possibly involved in this process is of high interest. In Figure 1, we have depicted three subsets that we would like to highlight, namely regulatory T-cells (Tregs), regulatory B-cells (Bregs), and innate lymphoid regulatory cells (ILCregs). Tregs are cells with a strong regulatory capacity and are generally referred to as the CD4+CD25+ regulatory T cell subset. In cow’s milk allergic adults, it has been demonstrated that the percentage and function of CD4+CD25+ Tregs is intact [42]. Also, in cow’s-milk-allergic children, the presence of Tregs has been investigated. A study by Savilahti et al. showed that in the peripheral blood of allergic children, CD4+CD25+ Tregs can be detected and are functionally active [43]. Therefore, specific immunotherapies aiming at the stimulation of these naturally occurring regulatory T cells may contribute to the re-establishment of clinical tolerance in a sustainable way in allergic individuals. 

One of the mechanisms by which CD4+CD25+ (Tregs) may enhance the process of tolerance induction is via the production of the suppressive cytokine IL-10. Results from a study investigating the cow’s-milk-specific T cell response in allergic children, which are tolerant to cow’s milk, show that their cow’s-milk-specific T cell response is dominated by the production of large amounts of IL-10 [44]. This suggests a key role for IL-10-producing T cells in a long-lasting tolerogenic reaction in individuals where the immune system is skewed toward an allergic phenotype. Another potential source of IL-10 is the subset of regulatory innate lymphoid cells. ILCregs have been recently described to be present in the gut and produce IL-10 and TGF-β upon pathogenic stimulation, thereby promoting a tolerogenic environment [45]. Although a direct suppressive effect of ILCregs on ILC2 cells, which produce IL-4 and have been shown to promote food allergy by enhancing mast cell activation and the disruption of Treg function [46] has not been demonstrated, the ILCreg-mediated production of IL-10 may play a role in the process of tolerance induction by affecting other immune cells. 

For B cells, it has been suggested that a subpopulation, called regulatory B cells (Bregs), is of importance for the induction and maintenance of tolerance to many different self- and non-self-antigens [47]. Whether Bregs play a role in CMA remains to be established. 

## 6. Conclusions

Important aspects to keep in mind when evaluating the different options for allergy prevention and the treatment of cow’s-milk-allergic individuals are the intended target population (infants, children, or adults) and the patient characteristics (IgE and IgG4 serum levels, SPT values). A better immunological understanding of the mechanism underlying a sustained tolerance development in cow’s-milk-allergic patients will aid in improving current therapies or developing new therapies.
